# Fusing Swarm Intelligence and Self-Assembly for Optimizing Echo State Networks

**DOI:** 10.1155/2015/642429

**Published:** 2015-08-04

**Authors:** Charles E. Martin, James A. Reggia

**Affiliations:** ^1^HRL Laboratories, LLC, 3011 Malibu Canyon Road, Malibu, CA 90265, USA; ^2^Department of Computer Science, University of Maryland, College Park, MD 20742, USA

## Abstract

Optimizing a neural network's topology is a difficult problem for at least two reasons: the topology space is discrete, and the quality of any given topology must be assessed by assigning many different sets of weights to its connections. These two characteristics tend to cause very “rough.” objective functions. Here we demonstrate how self-assembly (SA) and particle swarm optimization (PSO) can be integrated to provide a novel and effective means of *concurrently* optimizing a neural network's weights and topology. Combining SA and PSO addresses two key challenges. First, it creates a more integrated representation of neural network weights and topology so that we have just a single, continuous search domain that permits “smoother” objective functions. Second, it extends the traditional focus of self-assembly, from the growth of predefined *target structures*, to functional self-assembly, in which growth is driven by optimality criteria defined in terms of the performance of emerging structures on predefined *computational problems*. Our model incorporates a new way of viewing PSO that involves a population of growing, interacting networks, as opposed to particles. The effectiveness of our method for optimizing echo state network weights and topologies is demonstrated through its performance on a number of challenging benchmark problems.

## 1. Introduction

In this paper we demonstrate how two very different nature-inspired methodologies,* self-assembly* (SA) [1] and* particle swarm optimization* (PSO) [2], can be integrated to provide a novel and effective means of concurrently optimizing a neural network's weights and topology. Such an approach addresses two important challenges. The first challenge is finding a more integrated representation of neural network weights and topology, so that rather than having to search in both a continuous weight space and a discrete topology space, there is just a single, continuous search domain that permits “smoother” objective functions. The second challenge is extending the traditional focus of self-assembly research from the growth of predefined* target structures* to functional self-assembly, in which growth is driven by optimality criteria defined in terms of the quality or performance of the emerging structures on predefined* computational problems*.

Swarm intelligence systems, which consist of autonomous agents interacting in a simple and local manner, exhibit complex global behavior that emerges as a consequence of local interactions among the agents [3–10]. Researchers have created a wide range of new problem-solving algorithms inspired by nature and based on the governing principles of swarm intelligence [11–17]. Of particular importance to the research presented in this paper is the powerful and broadly applicable swarm intelligence based optimization method known as particle swarm optimization (PSO) [18–23].

Particle swarm optimization has been applied extensively to train neural network weights. A wide variety of different adaptations and hybridizations of PSO have been developed for this purpose [24–30]. Despite the success in using PSO to optimize network weights, there has been only limited success in applying it to topology optimization, and such applications have largely been restricted to feedforward networks. The methods that do exist implement fairly complicated adaptations of the basic PSO algorithm or enforce stringent restrictions on the domain of feasible network topologies [31–33]. While the method presented in this paper does not adapt the number of nodes in the network, as some other algorithms do, it optimizes over general, recurrent neural network topologies using one of the most basic forms of PSO, which is a unique feature of our approach. Furthermore, the form of the underlying model of network growth that we present here does not place any constraints on the type of PSO used to drive the optimization, and therefore the user may readily swap in whatever version of PSO is deemed most suitable for the learning task at hand. Our method's incorporation of basic PSO would be particularly advantageous in cases where the topology of a physical network was being optimized.

**Figure 1:**
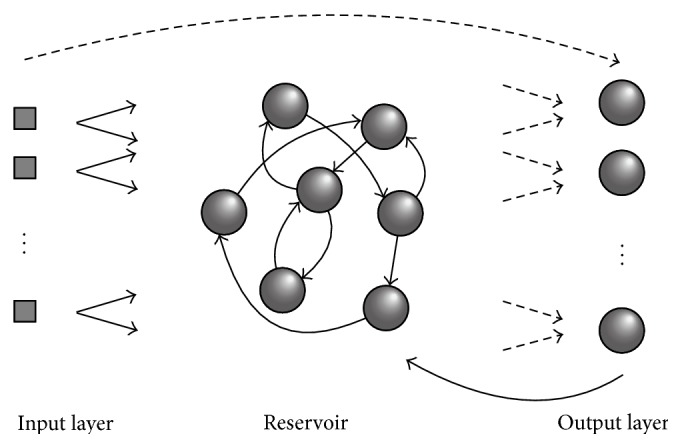
Schematic of an echo state network (ESN). The solid arrows represent connections with fixed weights, and the dashed arrows represent connections with trainable weights. Connections from the input layer to the output layer and from the output layer to the reservoir are optional. As usual, input to the network enters through the input layer and the network's output is generated in the output layer. The bias node and its connections with fixed weights to the reservoir are not shown.

The work presented here involves the optimization of a relatively recently developed class of recurrent neural network models known as* echo state networks* (ESNs) [34]. Echo state networks have already been successfully applied to a wide range of different problems [35–45]. They consist of an input layer, a hidden layer or “reservoir,” and an output layer (shown in Figure 1). Typically, each neuron in the input layer connects to every neuron in the reservoir; there is randomly generated sparse connectivity among reservoir neurons; each neuron in the reservoir connects to each neuron in the output layer; a bias neuron may connect to the neurons in the reservoir, and sometimes connections from the input layer to the output layer and from the output layer to the reservoir are present. The central innovation of the echo state network approach is that only the weights on the connections from the reservoir to the output neurons (output weights) are trained, and the activation functions of the output neurons are linear so all that is needed to train them is linear regression. The remaining weights are typically assigned random values. For an echo state network with *N*_*r*_ reservoir neurons and *N*_*o*_ output neurons, the output weights are trained as follows. A sequence of training data of length *L* + *M* is chosen, where *M* > *N*_*r*_. The first *L* values of the sequence are passed through the network in order to remove the effects of the initial state of the reservoir. Then, the remaining *M* values are input into the network, and the resulting reservoir states ${\vec{e}}_{i}\in {\mathbb{R}}^{N_{r}}$, for *i* = 1,…, *M*, are assigned to the rows of the matrix **S** ∈ *ℝ* ^*M*×*N*_*r*_^. For each network input, and resulting reservoir state ${\vec{e}}_{i}$, there is a target network output ${\vec{d}}_{i}$. The target network outputs are assigned to the rows of the matrix **D** ∈ *ℝ* ^*M*×*N*_*o*_^ such that the *i*th rows of **S** and **D** are the corresponding reservoir state and target output pair. Let **W** ∈ *ℝ* ^*N*_*r*_×*N*_*o*_^ be the output weight matrix, where the *j*th column of **W** represents the weights on the connections from the reservoir to the *j*th output neuron. Training the output weights amounts to finding an approximate solution **W**_*a*_ to the overdetermined system

(1) $$\begin{array}{c} SW=D. \end{array}$$

The output weights **W**_*a*_ are determined by solving (1) in a “least squares” sense.

Self-assembly involves the self-organization of discrete components into a physical structure, for example, the growth of connections between nodes in a physical network. Work in this area has traditionally focused on what we will refer to as the* classic self-assembly problem*, which entails the design of local control mechanisms that enable a set of components to self-organize into a given target structure, without individually preassigned component positions or central control mechanisms. Issues surrounding self-assembly have been a very active research area in swarm intelligence over the last several years, with recent work spanning computer simulations [46–51], physical robotics [52–57], and the modeling of natural systems [1, 12]. However, to the best of our knowledge, there has been no work on using swarm intelligence methods to extend the classic self-assembly problem to* functional self-assembly*, in which components self-organize into a computing structure that is optimized for a predefined computational problem.

The research presented in this paper is concerned with the self-assembly of neural network architectures. Unlike most past work on self-assembly, a major aspect of the work presented here involves the growth of connections between discrete, spatially separated nodes/components [58]. We recently demonstrated how swarm intelligence in the form of collective movements can increase the robustness of network self-assembly and enhance the ability to grow large, topologically complex neural networks [50]. However, this earlier work focused on the classic self-assembly problem where a target network was prespecified, which is in contrast to the more difficult problem of functional self-assembly that we consider here.

Other related past works in computer science and engineering, where research on artificial neural networks is concerned with application-related performance, have largely ignored the issues of neural network growth, development, and self-assembly, with two exceptions. First, a number of computational techniques have been created to optimize neural network architectures by adding/deleting nodes/connections dynamically during learning [59–63]. Unlike the approach taken here, these past network construction methods do not involve growth or self-assembly in a physical space and so are not considered further. Second, a technique known as* developmental encoding* has been used by researchers evolving neural network architectures with genetic algorithms/programming [64–70]. Unlike the work presented here, in these past techniques different individuals within a population do not directly interact with one another during the development process. Such interaction occurs only indirectly through the processes of selection and crossover.

Both weights and topology affect a neural network's performance. To date, substantially more focus has been placed on techniques for optimizing a neural network's weights as opposed to its topology (the number and connectedness of its nodes). One of the primary reasons for this discrepancy is that the space searched by an optimization method for a good set of weights (the “weight space”) is continuous. Thus a good set of weights can be found using one of a wide variety of powerful and well-studied optimization techniques based on local gradient information [71]. Additionally global optimization techniques such as particle swarm optimization (PSO), evolutionary computation (EC), and simulated annealing have proven to be very effective at weight optimization.

When optimizing in continuous domains, specifically multidimensional Euclidean space *ℝ* ^*n*^, optimization algorithms tend to operate under the basic heuristic (referred to here as the continuity-heuristic) that, given two points in a search space, each of which represents a good solution, it is likely that a better solution exists somewhere between or around these points. This heuristic has been found to be generally useful in optimizing objective functions defined on *ℝ* ^*n*^. The “topology space,” however, is a discrete space. The discrete nature of this search domain coupled with the intrinsic interdependence between neural network weights (parameters) and topology (structure) results in a variety of additional challenges not encountered when optimizing the weights of a network with a fixed architecture. First, it is common for many of the nodes in a neural network to be identical from a computational perspective, such as the nodes in the hidden layer, which means that many pairs of points in the topology space that are far apart will represent identical or very similar topologies. Second, certain connections may influence performance much more than others. This effect depends on factors such as a network's topology, the learning algorithm used, and the computational problem a network is tasked with solving. This means that in typical representations of the topology space, where a network that has an input-to-output connection would have a nearly identical representation to one that does not have such a connection (all other connections being the same), many points (topologies) that are near each other will represent network architectures that are associated with vastly different fitness values. Third, the quality of a particular topology is dependent on the set of weights associated with it and vice versa. This interdependence means that, rather than having a fixed fitness value, a point (topology) in the topology space has a distribution of fitness values generated by associating different sets of weights with its connections. This fact increases the “roughness” of the fitness landscape defined over the topology space. The first of the above characteristics implies that the distance between two points in the topology space often does not accurately reflect the similarity/dissimilarity of the topologies represented by the points. The second and third characteristics, coupled with the discrete nature of the topology space, imply that nearby points often represent topologies with very different fitness values, which produces very rough fitness/objective functions. Therefore, the properties that make the continuity-heuristic useful in the weight space are largely absent from the topology space.

If we could find a more integrated means of representing neural network weights and topology, such that the search domain consisted of a single, continuous “weight-topology space,” then this representation might preserve the continuity-heuristic and permit smoother objective functions. This is precisely what we have done. The integration involves* representing weights and topology using self-assembling neural networks that grow through a single, continuous three-dimensional space*. Our approach makes use of the fact that given two neural networks with different topologies, if the connections that these networks do* not* have in common have weights that are small enough in magnitude, then the networks will have roughly the same* effective* topologies in the sense that signals transmitted via these connections will be highly attenuated and thus tend to have very little influence on network dynamics. Therefore, from the perspective of network performance, it is as if the connections are not actually there. The idea of a neural network having an effective topology is a key concept in the work presented here. It explains why our approach can simultaneously optimize both weights and topology while operating over a continuous space. Specifically, as long as the weight threshold that triggers the removal (addition) of a connection is small enough, then traversing this threshold in the direction that results in the removal (addition) of a connection will yield a network with a new topology but which has nearly identical dynamics compared to its counterpart network. This is the case because the removed (added) connection would have a weight with magnitude near 0. Thus, if the objective function depends only on network dynamics, then this new network and its counterpart will evaluate to nearly identical values under the objective function.

The primary contributions of the work presented here arise from addressing the challenges inherent in the simultaneous optimization of neural network weights and topology. The first contribution is the development of a means of using network self-assembly as a representation of the search domain encountered in this optimization problem, thereby simplifying the domain to a single, continuous space. Second, our work puts forward a new way of viewing PSO that involves a population of growing, interacting networks, as opposed to particles. This adaptation is used to turn the self-assembly process into an optimization process and, to the best of our knowledge, is the first demonstration of such a complimentary relationship between self-assembly and particle swarm optimization. Third, the version of PSO that our work incorporates, which is a particularly elegant form of the algorithm, has not been previously used to simultaneously optimize neural network weights and topology. Lastly, we demonstrate the effectiveness of a software-based implementation of the integration of self-assembly and PSO by using it to grow high-quality neural network solutions to a variety of challenging benchmark problems from the domains of time-series forecasting and control.

## 2. Methods

In this section we introduce our model that represents an extension of the traditional self-assembly problem in that the growth of network structures is based on optimality criteria and not on target structures that are specified* a priori*.

### 2.1. Integrating Self-Assembly and Particle Swarm Optimization

We now present the details of our model for simultaneously optimizing neural network weights and topology. We call this model SINOSA, which stands for Swarm Intelligent Network Optimization through self-assembly. In this model groups of growth cones that belong to different networks simultaneously grow through the same three-dimensional space. During the growth process the growth cones from different networks interact with one another through a mechanism inspired by particle swarm optimization. Concurrently, the networks receive input derived from a computational problem that they must learn to solve. The combination of this interaction, and the activity run through the networks during the development process, leads to the self-assembly of neural networks with weights and topologies that are optimized for solving the problem at hand. An animation that exemplifies the growth process can be viewed at supplied URL (see Supplementary Material available online at http://dx.doi.org/10.1155/2015/642429).

#### 2.1.1. Objects and Relations

**Figure 2:**
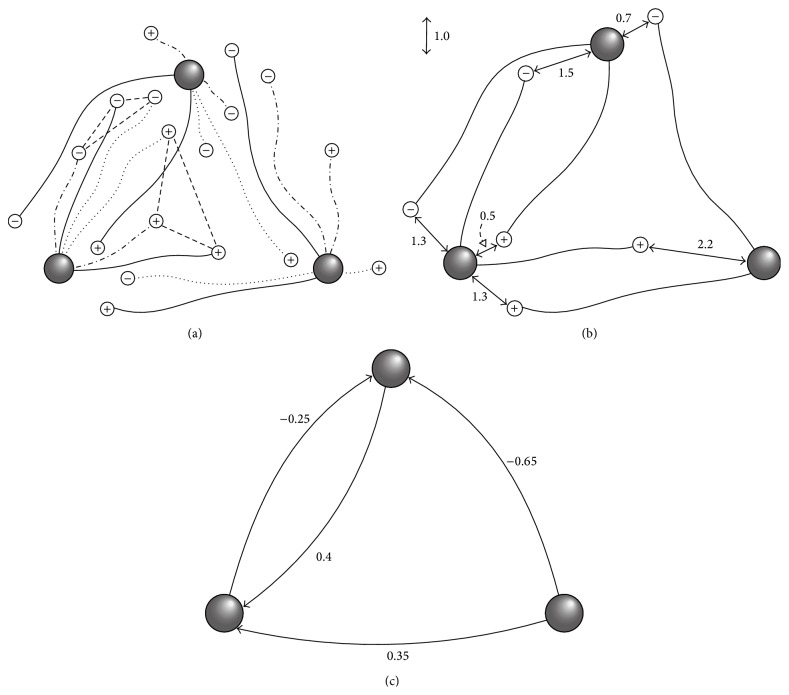
Three growing neural networks and their interpretations as static neural networks based on the SINOSA model. The three large spheres represent cells, the smaller circles represent growth cones, and the lines between cells and growth cones denote connections (axons). The growth cones that are drawn with a “+” symbol have a positive weight field, and those that are drawn with a “−” symbol have a negative weight field. (a) The growth cones and axons that belong to a particular growing network are all shown with the same line-style (solid, dotted, or dash-dot). The straight dashed lines between growth cones indicate two of the six growth cone neighborhoods. Growth cones within a neighborhood interact with one another according to the canonical PSO algorithm. All three growing networks share the same three cells. (b) The solid-line growing network is shown without the other two. (c) The corresponding static network to which the solid-line growing network is mapped based on the proximity of its growth cones to their target cells. The numbers represent connection weights.

Throughout this section the concrete example of the model illustrated in Figure 2 is referenced for clarification. The SINOSA model consists of a set of cells **C** with fixed positions in 3D space that are assigned* a priori*. The cells represent neuron cell bodies. Each cell *c*_*i*_ ∈ **C** has a set **N**_*c*_*i*__⊆**C**, which may be empty, of “neighbor” cells that it can connect to, where *i* = 1,2,…, |**C**|. In Figure 2 the three large grey spheres represent cells **C**, and each cell is allowed to connect to any other cell, including itself. Thus, **N**_*c*_*i*__ = **C**, for *i* = 1,2, 3.

Each growing network consists of the same set of cells **C** and a unique set of growth cones that guide the network's axons through the three-dimensional space. Given *n* simultaneously growing networks, each cell *c*_*i*_ has *n* sets of growth cones **G**_*ij*_, where *j* = 1,2,…, *n*. Any given cell *c*_*i*_ contributes the same number of growth cones to each growing network. That is, for all *j* and *ℓ*, |**G**_*ij*_ | = |**G**_*iℓ*_|, ensuring that all of the growth cone neighborhoods (explained below) among the growth cones in **G**_*i*_ = ⋃_*j*_ **G**_*ij*_ are of the same size. If **N**_*c*_*i*__ is empty, then so is **G**_*ij*_, for all *j*. The *j*th growing network gnet_*j*_ consists of the set of cells **C** and the set of growth cones **G**_*j*_ = ⋃_*i*_ **G**_*ij*_ that produce the network's growth. That is, gnet_*j*_ is defined by the ordered pair 〈**C**, **G**_*j*_〉. Because each growing network consists of the same set of cells **C**, they all have exactly the same number of growth cones (|**G**_*j*_ | = |**G**_*ℓ*_|, where *j*, *ℓ* = 1,2,…, *n*). In Figure 2 the small circles represent growth cones, and the lines that connect the cells and growth cones are axons. In this case there are *n* = 3 growing networks, each having six growing axons. The growing axons of any particular network are shown in a unique line-style (solid, dotted, or dash-dot). To clarify this, Figure 2(b) shows only the solid-line growing network.  Figure 2(c) shows the static network that is derived from the solid-line growing network, which is described in the next section.  Figure 2(a) illustrates how all three networks simultaneously grow through the same space and share the same three cells.

Let *c*_*k*_ ∈ **N**_*c*_*i*__ be the *k*th neighbor cell of *c*_*i*_. Then for each *c*_*k*_, the cell *c*_*i*_ contributes exactly two growth cones (*g*_*ijk*_^*s*^ for *s* ∈ {“ + ”, “ − ”}) to each of the growing networks *j* = 1,2,…, *n*. When *s* = “+” the growth cone represents positively weighted connections, and when *s* = “−” the growth cone represents negatively weighted connections. The positive-negative growth cone pair *g*_*ijk*_^+^ and *g*_*ijk*_^−^ may establish connections to exactly one target cell, namely, *c*_*k*_. Based on these relations and since **N**_*c*_*i*__ = **C** for *i* = 1,2, 3 in Figure 2, each of the cells contributes three positive-negative growth cone pairs to each of the three growing networks. However, for the sake of clarity only 6 of the 18 growing axons per network are shown.

#### 2.1.2. Interpreting Network Growth as Self-Assembly

The SINOSA model grows neural networks that, in their completed form, have fixed connections. Thus, it is necessary to interpret the positions of a growing network's growth cones relative to their target cells so as to map a* growing network *gnet_*j*_, to a* static network *snet_*j*_. In particular, if the positive-negative growth cone pair *g*_*ijk*_^+^ and *g*_*ijk*_^−^ from cell *c*_*i*_ and growing network gnet_*j*_ are both positioned so as to be able to establish a connection to cell *c*_*k*_, then the weight on the connection from *c*_*i*_ to *c*_*k*_ in the static network snet_*j*_ is the sum of the individual weights contributed by the growth cone pair.

In the SINOSA model the function from the space of growing networks to the space of static networks is designed around the need to create a neural network representation that more closely integrates the concepts of topology and connection weight so that the canonical PSO algorithm can be used to optimize these network characteristics effectively. This function is implemented as follows. Each growth cone is considered to be at the center of its own spherically symmetric “weight field” that is finite in extent, and its corresponding weight has a magnitude that decreases to zero as the distance from the growth cone increases. A growth cone establishes a connection with a target cell if the cell is within the boundary of its weight field; otherwise no connection is created. The spacing between cells is such that no more than one cell can be within a growth cone's weight field at any given time. The weight on the connection is the value of the field at the target cell's center. Formally, a* weight field* is a function from *ℝ* to *ℝ* with the form

(2) $$\begin{array}{c} w\left(\;r\;\right)=\left\{\begin{array}{cc} ar^{\alpha }+b, & \text{if}\;\;r<r_{0}\\ 0, & \text{if}\;\;r\geq r_{0}, \end{array}\right. \end{array}$$

where *a*, *b* ∈ *ℝ*, *r* ≥ 0 is the distance of the target cell from the center of the growth cone, *r*_0_ > 0 is the extent of the weight field, and *α* > 0. In the SINOSA model it is assumed that *w*(*r*) → 0 as *r* → *r*_0_. Thus *w*(*r*_0_) = *ar*_0_^*α*^ + *b* = 0, which implies that *a* = −*b*/*r*_0_^*α*^ = −*w*(0)/*r*_0_^*α*^. Figures 2(b) and 2(c) illustrate how one of the three interacting, growing networks, shown together in Figure 2(a), is mapped to a static network based on the weight field interpretation of the growth cones' positions relative to their target cells. The growth cones that are drawn with a “+” symbol have a positive weight field represented by the function

(3) $$\begin{array}{c} w_{+}\left(\;r\;\right)=\left\{\begin{array}{cc} -\frac{1}{2}r+1, & \text{if}\;\;r<2\\ 0, & \text{if}\;\;r\geq 2, \end{array}\right. \end{array}$$

where *r* is the distance between a growth cone and one of its target cells. The growth cones that are drawn with a “−” symbol have a negative weight field expressed by the function

(4) $$\begin{array}{c} w_{-}\left(\;r\;\right)=\left\{\begin{array}{cc} \frac{1}{2}r-1, & \text{if}\;\;r<2\\ 0, & \text{if}\;\;r\geq 2. \end{array}\right. \end{array}$$

Thus, in this scenario the weights are restricted to the interval [−1,1]. Figure 2(b) shows the solid-line network's growing axons, along with the distance between each growth cone and its nearest target cell.  Figure 2(c) shows the static network derived from the solid-line growing network. The numbers here are the connection weights. This mapping occurs as follows. The cell shown in the lower left hand corner of Figures 2(b) and 2(c) establishes a connection with weight *w*_−_(1.5) = −0.25 to the upper cell but does not establish a connection with the cell in the lower right hand corner because *w*_+_(2.2) = 0. The upper cell makes a connection to the lower left hand cell with weight *w*_+_(0.5) + *w*_−_(1.3) = 0.4. The lower right hand cell makes a connection to the lower left hand cell with weight *w*_+_(1.3) = 0.35, and it makes a connection to the upper cell with weight *w*_−_(0.7) = −0.65. The other two growing networks are mapped to their corresponding static network representations in an analogous manner.

The function that maps growing networks to static networks is formulated so that a small change in the position of a growth cone produces a small change in the weight on a connection, or if the change in position results in the addition or removal of a connection, then the added or removed connection has a weight that is small in magnitude. In other words, a small change in the physical configuration of a growing network will produce a small change in the weights and topology of the static network to which it is mapped. This characteristic, coupled with the fact that network optimization via the SINOSA model occurs in a single, continuous weight-topology space, results in much smoother objective functions.

#### 2.1.3. Incorporating PSO

Using network self-assembly as our representation scheme yields a single, continuous weight-topology space that needs to be searched during the optimization process. In other words, we need to extend the classic self-assembly problem to functional self-assembly. Given that we have a single, continuous search domain, a wide variety of different optimization algorithms could be used for this purpose. However, we chose to use the PSO algorithm because it is intuitive and effective, and our model incorporates a version of the algorithm for which application to the concurrent optimization of neural network weights and topology has not previously been explored. Specifically, we utilize one of the most basic formulations of PSO, which will be referred to as* canonical* PSO. Canonical PSO specifies that the particles velocities are governed by

(5) v→it+1⟵χv→it+apu→1⊗p→best,i−r→i+anu→2⊗n→best,i−r→i,

where ${\vec{r}}_{i}(t)$ is the position of the *i*th particle at time *t*, ${\vec{v}}_{i}(t)$ is its velocity, ${\vec{p}}_{best,i}$ is the current best position of the *i*th particle, ${\vec{n}}_{best,i}$ is the best position among any of its neighbor particles, *χ* is a scaling factor known as the constriction coefficient, *a*_*p*_ and *a*_*n*_ are positive constants, ${\vec{u}}_{1}$ and ${\vec{u}}_{2}$ are vectors whose components are drawn from the uniform probability distribution over the unit interval, and the ⊗ symbol represents the component-wise vector product (i.e., [*a*_1_ *a*_2_]⊗[*b*_1_ *b*_2_] = [*a*_1_ *b*_1_ *a*_2_b_2_]). It is standard practice to update the positions of the particles using a Forward Euler step with a step-size of 1.0; that is, ${\vec{r}}_{i}(t+1)\leftarrow {\vec{r}}_{i}(t)+{\vec{v}}_{i}(t+1)$. The appeal of this version of PSO lies in its simplicity and in its proven effectiveness on a wide range of optimization problems.

In order to integrate particle swarm optimization and self-assembly the particles in PSO are viewed as being part of a larger structure. Almost all implementations of PSO consider the* particle* to be the fundamental type of object capable of movement and interaction during the optimization process. In the research presented here, the growing* network* plays the role of the fundamental type of object involved in the optimization process. That is, instead of a population of moving particles, there is a population of growing networks. The transition from particles to networks is achieved by having* growth cones* play the role that* particles* do in traditional PSO. Growth cones occur at the leading tips of growing axons (connections) and guide their movements through the physical space. The growth cones' movements are dictated by the canonical PSO equation (5), and because growth cones occur at the leading tips of growing axons, their movements generate network growth. Unlike in traditional PSO, the position of a growth cone (particle), however it is interpreted, is only meaningful when the axon/neuron that it is a part of is taken into account.

Since the growth cones from different growing networks interact with one another according to the canonical PSO algorithm, during the self-assembly process each growth cone must be assigned a quality (fitness) value that indicates the usefulness of the best solution component (connection) the growth cone has found, and it must remember its personal best position, which represents the best connection found by the growth cone up to the current point in the growth process. Specifically, at each discrete time-step *t* ∈ *ℕ* the performance of each static network snet_*j*_(*t*) on some set of training data is determined, where *j* = 1,2, .., *n*. For each growing network gnet_*j*_(*t*), if the performance of snet_*j*_(*t*) is better than the performance of snet_*j*_(*t* − *τ*) for all *τ* ∈ *ℕ* such that 0 < *τ* ≤ *t*, then the fitness value of gnet_*j*_(*t*), or more specifically its growth cones *g*_*ijk*_^*s*^ ∈ **G**_*j*_, is set to the performance value of snet_*j*_(*t*), and the personal best position of each growth cone *g*_*ijk*_^*s*^ is set to its current position. In theory, it is possible to determine the fitness of each growth cone in a network individually, rather than collectively at the network level. To do this one would need to determine a fitness value proportional to *𝔼*[Network  Performance∣Growth  Cone  Weight], the expected value of the network's performance as a function of the growth cone's weight. We chose the former approach for two reasons. First, computing such an expectation value requires averaging network performance over a very large number of possible growth cone positions that constitute instantiations of different networks. Second, each individual connection has only minimal influence on network performance, and thus optimizing them individually tends to lead to convergence on suboptimal solutions.

Any growth cone *g*_*ijk*_^*s*^ must have a set of neighbor growth cones **N**_*g*_*ijk*_^*s*^_ that influence its movements. As in most implementations of PSO, the research presented here adheres to the condition that the neighbor relation is symmetric. That is, if *g*_*iℓk*_^*s*^ is a neighbor of *g*_*ijk*_^*s*^, then *g*_*ijk*_^*s*^ is a neighbor of *g*_*iℓk*_^*s*^. There is a wide variety of different ways that a growth cone's neighbors could be selected. However, certain characteristics of the self-assembly/optimization process limit the number of useful choices. It is an underlying assumption of the PSO algorithm that the closer two neighbor particles get to one another, the more similar the solutions or solution components that their positions represent are. It is essential for the effectiveness of the PSO algorithm that if two growth cones *g*_*ijk*_^*s*^ and *g*_*iℓk*_^*s*^ are neighbors, and they occupy the same position, then they represent exactly the same weighted connection in their respective static networks snet_*j*_ and snet_*ℓ*_.

In the SINOSA model, if two growth cones occupy the same position but are guiding axons from different cells, then they represent two completely different connections (solution components). Likewise, if two growth cones occupy the same position but do not have exactly the same set of target cells, then they may represent different connections. These two scenarios, and the need for growth cones that are neighbors to avoid circumstances where they occupy the same position and yet represent different weighted connections, lead to three necessary growth cone neighborhood properties. First, if a pair of growth cones are neighbors, then they must be guiding axons from the same cell. Second, if a pair of growth cones are neighbors, then they must have exactly the same set of target cells. Third, if a pair of growth cones are neighbors, then their weight fields must be expressed by the same function. The following is a simple and effective way of choosing a growth cone's neighbors such that these properties are satisfied. For any cell *c*_*i*_ and growing network gnet_*j*_, the neighbor growth cones of the growth cone *g*_*ijk*_^*s*^ ∈ **G**_*ij*_ with target cell *c*_*k*_ and sign *s* are members of the set **N**_*g*_*ijk*_^*s*^_ ⊂ {*g*_*iℓk*_^*s*^ ∈ **G**_*iℓ*_∣*ℓ* = 1,2,…, *n*}. In Figure 2(a) the dashed lines between growth cones explicitly show two of the 18 growth cone neighborhoods (only 6 of the 18 growing axons per network are shown). Because each growth cone neighborhood consists of three growth cones connected in a ring topology, **N**_*g*_*ijk*_^*s*^_ = {*g*_*iℓk*_^*s*^ ∈ **G**_*iℓ*_∣*ℓ* = 1,2, 3∧*ℓ* ≠ *j*}.

When the SINOSA model is used to grow a network that is optimized for a computational problem, on every time-step of the growth process, the performance of each static network is evaluated and used to update the fitness values of the growth cones. The positions of the growth cones are then updated according to the canonical PSO algorithm. Then, on the next time-step, the new physical configurations of the three growing networks are mapped to their corresponding static networks, and the evaluation process repeats. The growth process terminates, and the best performing static network found during the growth process is returned, after a predefined number of time-steps, or once one of the static networks satisfies a prespecified performance criterion.

### 2.2. Experimental Methods

Here, we cover the implementation details of the SINOSA model when it is used to optimize neural networks for the Mackey-Glass time-series forecasting problem and the double pole balancing problem. These problems were selected because they are challenging and widely used benchmark tasks in the domains of time-series forecasting and control, and a wide variety of different neural network training/optimization algorithms have been used in solving them.

#### 2.2.1. Computational Test Problems

**Figure 3:**
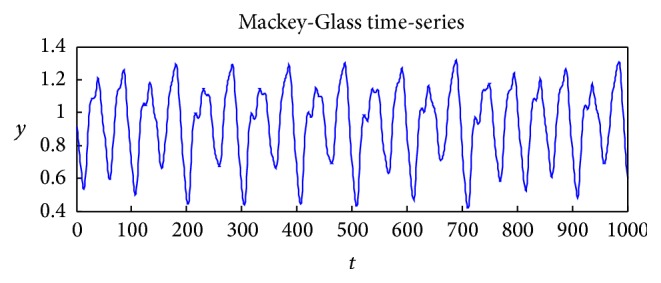
An example of the time-series generated by (6) with parameters *α* = 0.2, *β* = 10.0, *γ* = 0.1, and *τ* = 17.

The first problem is forecasting the chaotic Mackey-Glass time-series [39, 72, 73]. The time-series is generated by the delay differential equation

(6) $$\begin{array}{c} \frac{dy}{dt}=\frac{\alpha y\left(t-\tau \right)}{1+y{\left(t-\tau \right)}^{\beta }}-\gamma y\left(\;t\;\right), \end{array}$$

where *α*, *β*, *γ*, and *τ* ∈ *ℝ* ^+^. When *τ* > 16.8 the time-series is chaotic.  Figure 3 shows a sample of the time-series.

**Figure 4:**
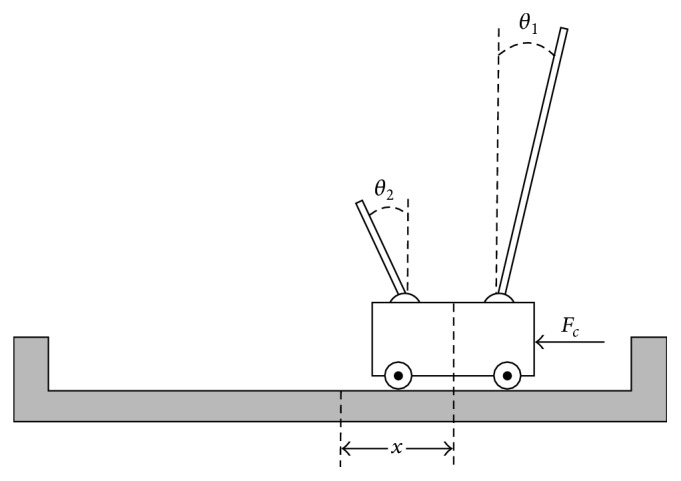
The cart-pole system used in the double pole balancing problem. The state of the system is defined by the position *x* of the cart relative to the center of the track and the angular positions *θ*_1_ and *θ*_2_ of the large and small poles relative to the vertical. The control force *F*_*c*_ is applied to the side of the cart, in a direction parallel to the track.

The second problem is the double pole balancing problem (see Figure 4), which is a classic benchmark control problem, particularly for neural network controllers (neural controllers) [74–76]. The double pole balancing problem consists of using a controller to balance two poles with different lengths that are hinged to the top of a cart that moves along a track of finite length. The controller attempts to keep the poles up-right by applying a force *F*_*c*_ to either side of the cart in a direction parallel to the track. To be successful, the controller must keep the cart within a specified distance *x*_limit_ from the center of the track, and it must keep each pole within a specified angular limit *θ*_limit_ from the vertical. The equations governing the dynamics of a cart with *N* poles can be found in [76].

#### 2.2.2. Implementation Details

The SINOSA model is implemented as a simulation environment written in Java. The computational experiments presented in Section 3 each ran on a computer with two quad-core 2.33 GHz Intel Xeon processors, 8 GB of shared RAM, and 12 MB of L2 cache per processor. The computational requirements listed here are for the growth of echo state networks using collective movements, unless stated otherwise. The environment in which networks grow was unbounded and no particular units were assigned to time and distance. The components of growing networks (cells, axons, and growth cones) were not able to collide with one another. The cells' positions remained fixed throughout the growth process. Unless stated otherwise, in every experiment the positions of the cells were fixed on a centered rectangular lattice with 8.0 distance-units between adjacent lattice points; there were 16 growing networks, and the growth cone neighborhoods adhered to a von Neumann topology (square lattice with periodic boundary conditions).

The dynamics of the growth cones are governed by the canonical PSO equation (5), where *a*_*p*_ = *a*_*n*_ = 2.0, *χ* = 0.65 for the Mackey-Glass experiments, and *χ* = 0.725 for the double pole balancing experiments. For all of the experiments, each growth cone's weight field was linear (*α* = 1) and had a radius *r*_0_ = 2.0. By convention, one time-step in the SINOSA model is equivalent to 1.0 unit of time.

Many of the experimental results presented in Section 3 are compared to* control cases* that incorporated random growth cone movements, as opposed to collective movements driven by the canonical PSO equation, generated as follows. At every time-step of the growth process each growth cone is placed at a unique, randomly selected position that is less than a distance *r*_0_ from the center of its target cell, where *r*_0_ is the extent of the growth cone's weight field. This way, the weights on the connections are randomly generated.

For each of the computational problems discussed in Section 2.2.1, the SINOSA model was used to grow echo state networks as solutions. The computational experiments described in the Results were designed to test the optimization capabilities of the SINOSA model.

## 3. Results

### 3.1. Mackey-Glass Time-Series

In all of the experiments that involve the Mackey-Glass time-series the parameters of (6) were set to the following commonly used values: *α* = 0.2, *β* = 10, *γ* = 0.1, and *τ* = 17. These values yield a “mildly” chaotic time-series. The time-series was generated by solving (6) using the Matlab delay differential equation solver* dde23* with a maximum step-size of 1.0, a relative error tolerance of 10^−4^, and an absolute error tolerance of 10^−16^. For every time-series generated from (6), an initial sequence of data points was randomly generated from the uniform probability distribution over the interval [0,1], and (6) was integrated for 1000 time-steps before collection of the time-series data began. This initial run-off period was necessary to remove the effects of the randomly generated initial condition. Consecutive data points in the sequences generated by the Mackey-Glass system were separated by 1.0 units of time. Data from the Mackey-Glass system was made more appropriate for processing by neural networks by mapping it into the interval [−1,1] using the hyperbolic-tangent function tanh(*x*) = (*e* ^2*x*^ − 1)/(*e* ^2*x*^ + 1). Network output was mapped back to the original range using the inverse of tanh(*x*) for testing, validation, and analysis. Each reservoir neuron used *f*(*x*) = tanh(*x*) as its transfer function and had an internal state governed by the leaky integrator differential equation [34]. The output neuron used the linear transfer function, *f*(*x*) = *x*, and did not have an internal state.

The ESNs grown for the Mackey-Glass time-series consisted of a single input neuron, a bias neuron, a single output neuron, and 50 neurons in the reservoir. The growth cones were permitted to establish connections from the input neuron to the reservoir neurons, from the bias neuron to the reservoir, and from the reservoir back to the reservoir. Additionally, each reservoir neuron had a permanent connection to the output neuron because the weights on the reservoir-to-output connections were derived using linear regression. The “Echo State Property,” which is a necessary condition on the reservoir dynamics for achieving good performance and in past work has typically been attained by manually scaling the spectral radius of the reservoir weight matrix to be less than one [34], was derived entirely through the network growth process.

The input neuron's set of neighbor neurons was the entire reservoir, as was the case for the bias neuron. Each reservoir neuron's set of neighbor neurons consisted of 5 randomly selected neurons in the reservoir. The output neuron did not have any neighbor neurons, because it did not have any growing axons. For each one of the 16 simultaneously growing networks, each neuron contributed one positively weighted growth cone (*b* = 1 in (2)) and one negatively weighted growth cone (*b* = −1), per neighbor neuron. Each of these positive-negative growth cone pairs had the same, single target neuron.

On every time-step each growing network was mapped to the static network represented by its current physical configuration. Before applying input to a network, the internal state and/or output of each neuron was set to zero. For each static network, the weights on the connections from the reservoir neurons to the output neuron were trained using teacher-forcing [39] with a sequence generated from the Mackey-Glass system that had 2100 data points. The first 100 data points of this sequence were fed into a network prior to any training with the purpose of removing the effects of the initial state of the reservoir. The next 2000 data points were then fed into the network, and linear regression in the form of the least squares method was performed between the resulting reservoir states (activities of the reservoir neurons) and the desired network outputs. The topology and weights of the connections from the input neuron to the reservoir, from the bias neuron to the reservoir, and from the reservoir neurons back to the reservoir were determined by the growth process.

The performance of an ESN on the data used to train the output weights is typically not a good measure of the network's ability to generalize to new data [77]. Thus, during the growth process the generalization performance of each static network was computed on every time-step and used to update the fitness values of the growing networks. The generalization performance measure was the normalized root mean square error computed over a set of 84-step predictions (NRMSE_84_) [34]. Specifically, on every time-step, after training the output weights, the NRMSE_84_ was computed for each static network on a group of 20 randomly selected sequences from the Mackey-Glass system. Each of these sequences consisted of 184 data points. In order to prevent overgeneralization, every 10 time-steps a new set of 20 sequences was randomly pulled from a pool of 200 different sequences. At the end of the growth process, which lasted for 3600 time-units, the best positions of the growth cones in each growing network represent the best performing networks found over the course of the entire growth process. There is one best performing network per growing network and it is instantiated by translating the best positions of the network's growth cones into the corresponding static network. The performances of the best static networks were validated by computing the NRMSE_84_ of each network using 100 new sequences, each of a length of 2084. The best performing network on this validation data was taken as the solution. Towards the end of a growth process the growing networks tend to converge on a particular configuration and this final validation step ensures that the solution network has the best* generalization* performance.

For the SINOSA model, 37 trials were run with collective movements generated by canonical PSO, and 38 trials were run with random movements. In the Mackey-Glass experiments, on average, one epoch of growth (explained in Section 3.1) of an ESN with 50 neurons in its reservoir requires 2 hours of CPU time. One epoch of growth of an ESN with 100 neurons in its reservoir requires 5 hours of CPU time. One epoch of growth of an ESN with 400 neurons in its reservoir requires 3 days of CPU time. The networks grown using collective movements have a mean NRMSE_84_ that is 68% smaller than those grown with random movements with 95% confidence.

Once the growth process had finished (after 3600 time-units) the grown networks were further optimized by refining the search process and running it for an additional 3600 time-units. This refinement was implemented by continuing the growth (search) process with new growth cones that had weight fields that were smaller in maximum magnitude. Specifically, for each connection in the best performing static network found during the first epoch, except the connections from the reservoir to the output neuron, a fixed connection with the same weight was created between the corresponding cells in the set **C**. When a static network was instantiated from a growing network during the second epoch, the weights on the connections in the static network were the sum of the weight values contributed by the growth cones and the fixed connections.

The network growth process generated by the SINOSA model was extended by one epoch, for a total of two epochs of growth.  Table 1 compares the results obtained using collective movements, with those obtained using random movements, when the growth process was extended. Each numeric value represents the mean NRMSE_84_ and the 95% confidence interval for the corresponding epoch of growth and class of movements. It can be seen that, for both collective and random movements, there is a small but statistically significant reduction in the mean NRMSE_84_ with each epoch of growth. Furthermore, for each epoch, the mean NRMSE_84_ of the networks grown using collective movements is smaller than that of the networks grown using randomly generated movements.

Further evidence of the effectiveness of the SINOSA approach can be gained through comparison with the studies presented in [34, 39], which represent state-of-the-art performance for Mackey-Glass time-series prediction. In [34], echo state networks with 400 neuron reservoirs were optimized to forecast the Mackey-Glass time-series (*τ* = 17.0). The best performing of these networks, which was hand-designed by an expert, had an NRMSE_84_ of 2.8 · 10^−4^. In [39], using the same parameter values for the Mackey-Glass time-series as used here, an echo state network with a 1000-neuron reservoir was hand-designed by an expert that had an NRMSE_84_ of 6.3 · 10^−5^. The SINOSA model was used to grow echo state networks with 400 neurons in their reservoirs to forecast the Mackey-Glass time-series. The grown networks produced an average NRMSE_84_ of 3.86 · 10^−5^, and the best of these networks had an NRMSE_84_ of 2.73 · 10^−5^. On average, the grown networks with 400 neurons outperformed the best hand-designed 400-neuron ESN by about an order of magnitude, and they also performed better than the 1000-neuron ESN. These results provide strong evidence of the effectiveness of using the SINOSA model to grow echo state networks, as opposed to the standard approach of optimizing them through trial and error.

### 3.2. Double Pole Balancing Problem

In all of the experiments that dealt with the double pole balancing problem, the parameters were set to the most commonly used values [75] as follows: mass of the cart *m*_*c*_ = 1 kg, mass of the 1st pole *m*_1_ = 0.1 kg, mass of the 2nd pole *m*_2_ = 0.01 kg, coefficient of friction between the cart and the track *μ*_*c*_ = 5 · 10^−4^ Ns/m, coefficients of friction between the poles and their hinges *μ*_1_ = *μ*_2_ = 2 · 10^−6^ Nms, length of the 1st pole *l*_1_ = 0.5 m, and length of the 2nd pole *l*_2_ = 0.05 m. The control force was restricted to the interval *F*_*c*_ ∈ [−10 N, 10 N]. The parameters defining the domain of successful control were set to *x*_limit_ = 2.4 m and *θ*_limit_ = 36°. As is the case in most past work, the equations governing the dynamics of the system were solved numerically using a fourth-order Runge-Kutta method with a step-size of 0.01 s. During a simulation, a portion of the state of the cart-pole system was given to a neural controller every 0.02 s, at which point the control force was updated. In the experiments presented here, a neural controller was not given velocity information as input; rather, it only received the current positions of the cart and two poles (*x*, *θ*_1_, and *θ*_2_). This was done in order to make the task of controlling the cart more difficult. These values were scaled to be in the interval [−1,1] prior to being input into a neural controller. This was done so that the values were in a range that was more appropriate for processing by neurons with hyperbolic-tangent transfer functions. The network output (control signal), which was in the interval [−1,1], was multiplied by 10.0 N in order to produce the control force. Reservoir neurons and the output neuron used the hyperbolic-tangent function as their transfer function. None of the neurons had an internal state.

The SINOSA model was used to grow echo state networks as controllers for the double pole balancing problem. These networks had three input neurons, one for each type of information; the network was given regarding the state of the cart-pole system (cart position, position of pole #1, and position of pole #2). The reservoir always consisted of 20 neurons. One output neuron was present, which produced the control signal. No bias neuron was used due to the symmetry of the cart-pole system. The growth cones were permitted to establish connections from the input neurons to the reservoir and from the reservoir neurons back to the reservoir. Additionally, each reservoir neuron had a permanent connection to the output neuron. The weights on the reservoir-to-output connections were fixed and drawn randomly with uniform probability from the interval [−30,30]. The network architecture was otherwise identical to that of the Mackey-Glass network (see third paragraph of Section 3.1) except that in this case each reservoir neuron had only 4 neighbor neurons.

During the growth process the performance of each static network was computed on every time-step. The function *f*_pole_ was evaluated to determine the performance of the echo state networks grown as controllers for the double pole balancing problem and is given by

(7) $$\begin{array}{c} f_{pole}=10^{-4}n_{I}+0.9f_{stable}+10^{-5}n_{II}+30\frac{n_{S}}{625}. \end{array}$$

Equation (7) was introduced in [75] and is based on performance (fitness) functions presented in past works on the double pole balancing problem. To compute the first term in (7) the cart-pole system is set to the initial state $\left(\theta_{1}(0)=4.5^{\circ },{\dot{\theta }}_{1}(0)=\theta_{2}(0)={\dot{\theta }}_{2}(0)=x(0)=\dot{x}(0)=0\right)$. The network is then allowed to control the system for up to 1,000 time-steps. The number of time-steps *n*_*I*_ that the controller keeps the cart and poles in the success domain (*x* ∈ [−2.4 m, 2.4 m] and *θ*_1_, *θ*_2_ ∈ [−36°, 36°]) is counted. If the system leaves the success domain at any time prior to time-step 1,000, then the simulation stops. The second term is a measure of the stability of the system during the last 100 time-steps while under neural network control and is expressed by the function

(8) $$\begin{array}{c} f_{stable}=\left\{\begin{array}{cc} 0, & n_{I}<100\\ \frac{0.75}{\sum_{i=n_{I}-100}^{n_{I}}\rho_{i}}, & n_{I}\geq 100, \end{array}\right. \end{array}$$

where

(9) $$\begin{array}{c} \rho_{i}=\left(\;\left|\;x\left(\;i\;\right)\;\right|+\left|\;\dot{x}\left(\;i\;\right)\;\right|+\left|\;\theta_{1}\left(\;i\;\right)\;\right|+\left|\;{\dot{\theta }}_{1}\left(\;i\;\right)\;\right|\;\right). \end{array}$$

The third and fourth terms are measures of a neural controller's ability to generalize. If *n*_*I*_ = 1000 after computing the first term, then the neural controller is allowed to control the system for up to additional 100,000 time-steps. The number of additional time-steps *n*_*II*_ that the controller keeps the cart and poles in the success domain is counted, and the simulation stops if the system leaves the success domain or *n*_*II*_ = 100,000. The fourth term is computed by putting the cart-pole system in 625 different initial conditions and allowing the network to control it for up to 1,000 time-steps from each starting configuration. The variable *n*_*S*_ represents the number of different initial conditions from which the neural controller was able to keep the system in the success domain for 1,000 consecutive time-steps. The 625 unique initial conditions are defined in [74].

On every time-step of the growth process each growing network was mapped to the static network represented by its current physical configuration so that its performance could be computed by evaluating (7). Before applying input to a network the output of each neuron was always set to zero. Before a network was permitted to control the cart and poles the dynamics of the cart-pole system were evolved for 0.2 s, and the resulting sequences of 10 system states were input into the network. The neural network growth process lasted for 600 time-units, after which the static network with the best performance (largest value of *f*_pole_) was taken as the solution.

A total of 51 trials were run starting from different, randomly generated initial conditions. In the double pole balancing experiments 200 time-steps of growth require approximately 0.3 hours of CPU time, 400 time-steps require 1.4 hours, and 600 time-steps require 3.3 hours.  Table 2 compares the performance of networks grown using collective movements to the performance of networks grown using random movements. The comparison of performance is made every 200 time-steps during the growth process. Each of the numeric values in the tables is shown with its 95% confidence interval. The values in Table 2 were computed as follows. For each trial, and at each of the three predefined time-steps (200, 400, and 600), two measures of the best performing network at that point in the growth process were recorded. The first measure was whether or not the network succeeded in achieving *n*_*II*_ = 100,000 when computing (7). The second measure was the value of *n*_*S*_. In Table 2 the term Measure_*II*_ refers to the fraction of best performing networks that achieved *n*_*II*_ = 100,000. The term Measure_*S*_ refers to the average value of *n*_*S*_ taken over all of the best performing networks. From these results it is clear that the networks grown with collective movements vastly outperform those grown with randomly generated movements on both performances measures.

A study that lends itself to comparison is presented in [75], which represents state-of-the-art performance on the double pole balancing problem. In this case echo state networks were optimized as controllers for the double pole balancing problem via a state-of-the-art form of evolutionary strategies that uses covariance matrix adaptation (CMA-ES). In this study CMA-ES was used to optimize the output weights and the spectral radius of the reservoir weight matrix. The experiments discussed in this section, in which the SINOSA model was used to grow ESNs as controllers for the double pole balancing problem, adhered to the same experimental setup and methods used in [75], except that in our study the grown neural controllers received only 10 inputs from the cart-pole system prior to beginning control instead of 20. Because evaluating the fitness/performance of the networks during the optimization process is the computational bottleneck, the number of such evaluations during an optimization run is a good measure of the overall computational cost of the process. On average it required 19,796 evaluations for the CMA-ES approach to find a neural controller capable of successfully controlling the cart for at least 200 out of the 625 initial configurations (the average was 224), and of these networks 91.4% were able to successfully control the cart for the additional 100,000 time-steps when it was started in the standard initial configuration. These results are very good with respect to past work on the double pole balancing problem. The SINOSA model was able to grow much better performing neural controllers and at much less computational expense. After only 9600 evaluations, on average, the best performing grown networks were able to successfully control the cart for 478 of the 625 initial configurations, and of these networks 100% of them were able to successfully control the cart for the additional 100,000 time-steps.

## 4. Discussion

The SINOSA model incorporates an integrated representation of a network's weights and topology. The objects in this representation are cells (neurons), axons, and growth cones. The cells have fixed positions, but the growth cones are able to guide the cells' axons through a continuous, three-dimensional space, producing a mature network with fixed connections and weights. As a result of the integrated representation, it is possible to incorporate the simplest, canonical form of PSO into the model for the purpose of simultaneously optimizing network weights and topologies. In effect, the SINOSA model treats the network self-assembly process as an optimization or search process, in which the simultaneous growth of multiple neural networks is driven by their interactions with one another and with problem related network input.

The ability of the SINOSA model to optimize neural networks for computational tasks was tested using two different very challenging and widely used benchmark problems from the domains of time-series forecasting and control. For each of the computational problems the echo state networks grown using collective movements generated via PSO outperformed those grown using randomly generated movements, and in most circumstances the performance gap was very large. Specifically, compared to the networks grown with random movements, those grown using the SINOSA model with collective movements performed 3 times better on the Mackey-Glass time-series forecasting problem and 19 times better and 12 times better on two generalization measures of the double pole balancing problem. Furthermore, the large improvements in network performance gained over random search come at very little additional computational cost because evaluation of network performance is the bottleneck.

Comparison with control cases that involve random search provides a base level of support for the optimization capabilities of the SINOSA model and demonstrates the feasibility of* functional self-assembly* as a means of network optimization. Further evidence of the effectiveness of the model at optimizing networks can be found by comparing the results presented here with studies that involve different methods of optimizing networks for the Mackey-Glass time-series forecasting problem and the double pole balancing problem. For example, the 400-neuron echo state networks grown using the SINOSA model perform nearly an order of magnitude better than the best performing 400-neuron ESN presented in [34] with the Mackey-Glass time-series. Furthermore, they even outperform the 1000-neuron ESN presented in [39] by an average factor of 1.6. As a point of further comparison, the networks grown via the SINOSA approach outperform those in [75] by an average factor of 2.1. Moreover, our ESNs were optimized with an average of 52% less computational expense. These results are also interesting in that they represent one of the comparatively small number of studies where echo state networks have been successfully trained as neural controllers using reinforcement learning.

It is worth pointing out that the SINOSA model can be cast in a more abstract representation by foregoing the self-assembly component. Imagine we are optimizing a network with *M* possible weighted connections. Then, according to (2) there are two distinct one-dimensional Euclidean spaces associated with each possible connection. Furthermore, there is a unimodal function *w*_+_(*r*) that is* nonnegative* and symmetric defined over the first space and a function *w*_−_(*r*) = −*w*_+_(*r*) that is defined over the second space. Each one of these spaces would contain a set of particles (growth cones) that was restricted to move within it. Only those particles within a given space would interact according to the PSO algorithm. A network would be created based on the positions of the particles in exactly the same manner described in Section 2.1.2. We chose to integrate self-assembly into our model from a desire to illuminate the processes by which physical networks might optimize their own weights and topology via self-assembly.

## 5. Conclusions and Future Directions

The concurrent optimization of neural network weights and topology is a challenging task due in large part to the roughness of the objective functions encountered when the search domain consists of both a continuous weight space and a discrete topology space. Through the SINOSA model we have demonstrated how network self-assembly can provide a useful means of representing this search domain in that the representation simplifies the domain to a single, continuous search space over which smoother objective functions can be defined. Furthermore, by using swarm intelligence in the form of collective movements to drive the network growth process, we were able to turn the self-assembly process into an optimization process.

The four primary contributions of our research are as follows:

- The SINOSA model constitutes a new and effective means of simultaneously optimizing the weights and topologies of neural networks. The model was used to grow echo state networks that performed substantially better on benchmark problems than networks optimized via random search. More importantly, the grown networks also outperformed the echo state networks presented in two different past studies, one in which the networks were hand-designed by an expert and the other in which they were optimized using a state-of-the-art form of evolutionary strategies (CMA-ES).
- There has been little past work on using PSO for the concurrent optimization of neural network weights and topology. The examples that do exist tend to involve fairly complicated adaptations of the method, significant constraints on permissible topologies, or hybridizations with other classes of methods such as evolutionary algorithms. In contrast, the SINOSA model uses the elegant canonical form of PSO to govern the growth/optimization process.
- In the vast majority of past work on PSO, the particles are embedded in a high dimensional abstract space, such as the domain of a function, they are the fundamental class of “objects” in the space, and the position of a particle represents a solution or solution component to the problem being solved. In contrast, the SINOSA model incorporates a novel way of viewing PSO in which growth cones (particles) are embedded in a continuous, three-dimensional space that is intended to model physical space, and* growing networks* are the fundamental class of objects in the space.
- Most past work on self-assembly has focused on the classic self-assembly problem, which entails the design of local control mechanisms that enable a set of components to self-organize into a* given* target structure. The SINOSA model represents an extension of the classic self-assembly problem to* functional self-assembly*, which includes the self-assembly of network structures with growth driven by optimality criteria defined in terms of the quality or performance of the emerging structures, as opposed to growth directed towards assembling a prespecified target structure.

There are a variety of potential future research directions for the SINOSA model. Here we mention three possibilities. First, it would be useful to extend this work to allow the number of neurons in the physical space to be able to increase or decrease during network assembly depending on the computational requirements of the problem being solved. Inspiration could likely be drawn from the fairly large number of past studies that involve dynamically modifying the number of nodes in a neural network. Second, further studies are needed to determine to what extent the parameters of the SINOSA model are problem dependent, and what values work well on a wide variety of different problems. Lastly, since its inception, the canonical form of particle swarm optimization has undergone a vast array of adaptations and hybridizations. Many of these enhancements could be incorporated into the SINOSA model without having to alter its fundamental constructs.

## Supplementary Material

This animation is an example of the network growth process governed by the SINOSA model. The small colored spheres are growth cones, which occur at the leading tips of growing connections (axons) and are responsible for guiding the connections from emitting to target cells (neurons). The large spheres represent the regions of space around cells that growth cones must enter in order for a connection to be established between a growth cone's emitting cell and the corresponding target cell. The weight on a connection established by a growth cone depends on the current position of the growth cone with respect to the center of the cell's “sphere-of-influence”. For clarity, only a small fraction of the total number of growth cones involved in the growth process are shown and the connections are not shown. There are seven different sets of growth cones shown, which are indicated by color. In this example each growth cone has a single target cell that it is allowed to establish connections with. The growth cones within a set all have the same target cell, and it is exactly these growth cones that interact with one another via a mechanism inspired by particle swarm optimization. Growth occurs in a three-dimensional space and the movements of the growth cones are not bounded. The growth process is initialized by placing each growth cone at a randomly selected location within the sphere-of-influence of its single target cell. Initially, the growth cones exhibit highly variable, wide-ranging movements as they explore different weighted connections or no connection at all. As the growth process progresses, and better networks are discovered, the growth cones exploit this information and begin to converge on single points in space, which ultimately represents a single best-performing network.

## Figures and Tables

**Table 1:** Mean NRMSE_84_ values on the Mackey-Glass time-series for networks grown with the SINOSA model.

Epoch	Collective movements	Random movements
1	5.89 · 10^−3^ ± 3.3 · 10^−4^	1.84 · 10^−2^ ± 8 · 10^−4^
2	4.98 · 10^−3^ ± 3.2 · 10^−4^	1.48 · 10^−2^ ± 7 · 10^−4^

**Table 2:** Performance values on the double pole balancing problem for networks grown with the SINOSA model.

Time-step	Collective, Measure_II_	Random, Measure_II_
200	0.667 [0.530, 0.780]	0.026 [0.005, 0.135]
400	0.961 [0.868, 0.989]	0.053 [0.015, 0.173]
600	1.0 [0.930, 1.0]	0.053 [0.015, 0.173]

Time-step	Collective, Measure_*S*_	Random, Measure_*S*_

200	372 ± 29	10 ± 5
400	462 ± 10	28 ± 15
600	478 ± 7	41 ± 17
